# Learning from algorithm-generated pseudo-annotations for detecting ants in videos

**DOI:** 10.1038/s41598-023-28734-6

**Published:** 2023-07-18

**Authors:** Yizhe Zhang, Natalie Imirzian, Christoph Kurze, Hao Zheng, David P. Hughes, Danny Z. Chen

**Affiliations:** 1 School of Computer Science and Engineering, Nanjing University of Science and Technology, Nanjing, 210094 China; 2 Department of Entomology and Department of Biology, Pennsylvania State University, University Park, PA 16802 USA; 3 Department of Bioengineering, Imperial College London, London, UK; 4 Institute for Zoology, University of Regensburg, Regensburg, DE Germany; 5 Department of Computer Science and Engineering, University of Notre Dame, Notre Dame, IN 46556 USA

**Keywords:** Computational models, Data processing, Image processing, Machine learning

## Abstract

Deep learning (DL) based detection models are powerful tools for large-scale analysis of dynamic biological behaviors in video data. Supervised training of a DL detection model often requires a large amount of manually-labeled training data which are time-consuming and labor-intensive to acquire. In this paper, we propose LFAGPA (Learn From Algorithm-Generated Pseudo-Annotations) that utilizes (noisy) annotations which are automatically generated by algorithms to train DL models for ant detection in videos. Our method consists of two main steps: (1) generate foreground objects using a (set of) state-of-the-art foreground extraction algorithm(s); (2) treat the results from step (1) as pseudo-annotations and use them to train deep neural networks for ant detection. We tackle several challenges on how to make use of automatically generated noisy annotations, how to learn from multiple annotation resources, and how to combine algorithm-generated annotations with human-labeled annotations (when available) for this learning framework. In experiments, we evaluate our method using 82 videos (totally 20,348 image frames) captured under natural conditions in a tropical rain-forest for dynamic ant behavior study. Without any manual annotation cost but only algorithm-generated annotations, our method can achieve a decent detection performance (77% in $F_1$ score). Moreover, when using only 10% manual annotations, our method can train a DL model to perform as well as using the full human annotations (81% in $F_1$ score).

## Introduction

**Figure 1:**
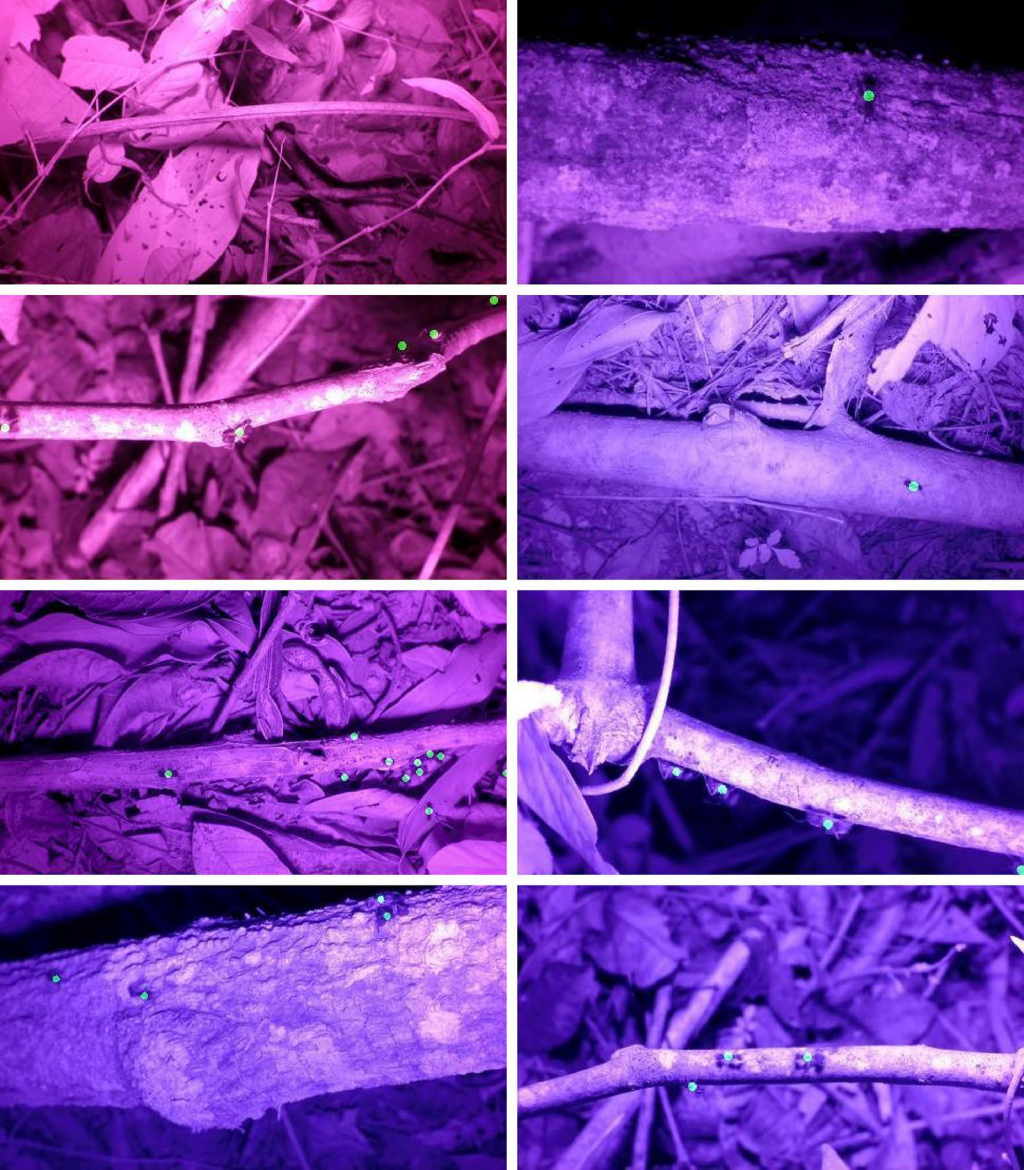
Image samples and the desired ant detection results (visualized in green colored disks). Please see the video attachment for video clip samples.

The studies of animal behaviors have changed with technical advances in video recording. Today, behavioral biologists use, for example, infrared or thermal cameras to capture unseen behaviors in the dark (e.g., the work^1–3^), high-speed cameras to record ultra fast movements in extreme detail (e.g., the work^4, 5^), and drones to study detailed movement patterns in videos (e.g., the work^6, 7^). These technical advances with individual marking made it also possible to explore the complex and fascinating life of social insects (i.e. ants, bees, termites and wasps) in detail (e.g., the work^8, 9^). social insects often live in highly dense groups which collectively make decisions. As they are easy to keep in the laboratory and experimentally manipulate them, they have become important study systems for example to learn about the evolution of sociality (e.g., the work^10, 11^), collective behavior (e.g., the work^9, 12^) and disease dynamics (e.g., the work^13–15^). Due to the steadily increasing amount of collected video footages (e.g., the work^1^), manual data analysis has become infeasible anymore (see Fig. 1). Modern deep learning (DL) based object detection methods are powerful tools for accurate detection of moving objects or animals and construction of their trajectories. But, such DL detection methods commonly require large amounts of annotated data for network training, which are time-consuming and labor-intensive to acquire.

**Figure 2:**
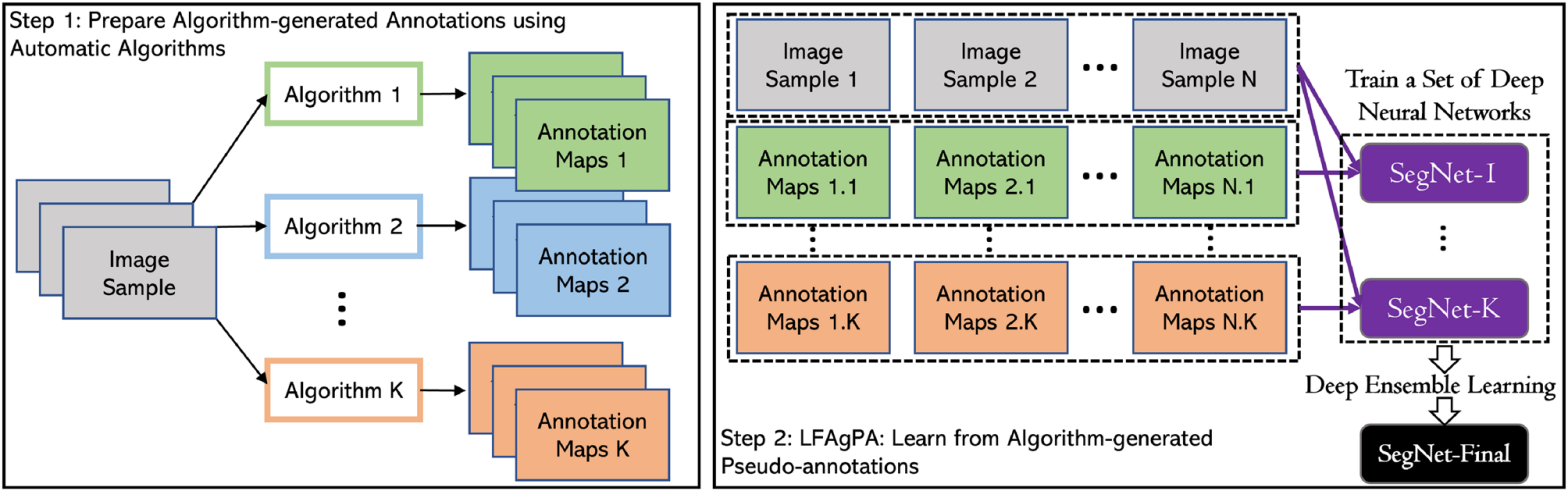
Schematic overview of our proposed learning framework. Step-1 generates pseudo-annotations for raw image data using foreground extraction algorithms. Step-2 trains DL based detection models using the generated training data from Step-1.

When biological videos are recorded in an environment where the background is relatively stable, automatic foreground extraction algorithms can be applied to extract preliminary foreground segmentation or detection results. Such preliminary detection results, although often noisy, contain valuable information on where the moving objects roughly are in the image frames of the videos. These results can be used as pseudo-annotations for training a DL based detection model. In this paper, we study how to build and train a DL detection model for ant video data using algorithm-generated pseudo-annotations. Our method has two major steps (see Fig. 2): (1) generate foreground objects using a (set of) reasonably good foreground extraction algorithm(s); (2) treat the results from step (1) as pseudo-annotations and use them to train a DL detection model.

*Our contributions* can be summarized as follows. (1) We demonstrate that it is practically feasible to utilize noisy algorithm-generated annotations in learning a deep neural detection model for processing biological video data. (2) A new probabilistic NN-Fit algorithm is developed to learn from networks trained with multiple annotation resources. (3) A hybrid learning framework is then proposed for learning a deep neural network using both the algorithm-generated annotations (noisy) and human-generated annotations (clean). With such a hybrid learning framework, a significant reduction in the human annotation is achieved to reach the same level of prediction performance.

In the following, we first describe the related methods and their relevance in “Related work” section. We then analyze essential mechanisms for learning from noisy labels in “Analyses on learning with noisy labels” section. Based on the analyses, we describe our proposed method in “Method” section. Finally, experiments are performed in “Experiments” section to validate our proposed method.

## Related work

### Object detection

Two-stage models (e.g., Faster R-CNN^16^ and Mask R-CNN^17^) and single-stage models (e.g., SSD^18^ and YOLO^19^) are common options for object detection problems. A two-stage model uses a Fully Convolutional Network^20^ (FCN) as its first stage for region proposal generation; and uses the proposed regions for segmentation or classification in the second stage. On the other hand, a single-stage model attains detection results using a group of encoding procedures (e.g., with a VGG like architecture^21^) and does not address any segmentation problem. In this paper, we demonstrate our new learning method using some popular DL models (U-Net^22^, DCN^23^) and Mask R-CNN^17^ for the ant detection problem.

### Pseudo-labels/annotations

Annotations for classification/detection/segmentation tasks usually refer to the image-/object-/pixel-wise ground truth provided by human experts. Pseudo-labels/annotations, on the other hand, are produced by a model or an algorithm. In the work^24^, pseudo-labels were proposed for semi-supervised learning for classification problems, where a DL model is trained using both images with manual labels and images with pseudo-labels produced by the current under-training DL model. It was shown that pseudo-labels generated by a DL model for unlabeled data could boost the performance of the under-training DL model^24^. Oliver et al.^25^ demonstrated that the method^24^ was still among the top performers for a semi-supervised learning classification problem using modern DL based models. However, there are some issues on using pseudo-labels generated by a under-explored DL model. First, if a DL model generates wrong predictions for some unlabeled images and such predictions are used to generate pseudo-labels, then the wrong ideas/concepts from the DL model could be further enhanced. Second, using pseudo-labels generated by a DL model still requires a certain amount of human-annotated images in the first place to initiate the model training (before it can generate pseudo-labels for unlabeled images). In contrast, our method uses algorithms to automatically generate pseudo-annotations (based on a common property of the ant videos) and requires zero manual annotation in the beginning. Further, our method is capable of using both human-annotated and algorithm-annotated training data. In experiments (“Scenario-1: using no human annotations” section), we directly compare our method with the method^24^ for the ant detection under the semi-supervised learning setting (Fig. 5).

### Self-supervised feature learning

Self-supervised feature learning is a relatively new research topic in the machine learning field. Pretext tasks (e.g., predicting the relative locations of two cropped image patches^26^, image colorization^27^, and solving jigsaw puzzles^28^) are hand-defined and algorithmically produced to train DL models to learn meaningful semantic features. MoCo^29^ and SimCLR^30^ are recent developments based on contrastive learning scheme. Although no manual labels are used, the learned feature representations from self-supervised learning methods do not output classification and detection predictions (additional training with manually labeled data is required for downstream tasks). In contrast, our method utilizes algorithm-generated annotations to directly train a DL model for a detection problem.

Lee et al.^24^ proposed to use pseudo-label for utilizing unlabeled data for training an image classification model. Pseduo-labels contain possible errors, and this pioneer work showed that training process of deep networks can reduce some of the errors via a self-correction mechanism. The self-correction mechanism was primarily demonstrated by experiments, but less theoretical studies were performed at that time. Pathak et al.^31^ proposed to learn visual representations by learning from unsupervised motion segmentation. Noisy segmentation maps were first generated by pixel grouping method based on motions in the videos, and these noisy segmentation maps were then used to train a new segmentation model. The trained networks can improve the quality of the segmentation from the original noisy segmentation and also learn meaningful feature representations. This phenomena was understood as that errors which are not systematic errors in the labels would be likely to be corrected by a learning process, due to the model’s limited capacity. Numerous work then followed to further study using pseudo-labels for utilizing unlabeled data in training a DL model.

### Learning from noisy labels

Learning from noisy labels has been a long-standing research problem in machine learning and computer vision. For a more thorough summary and analysis, one can refer to a recent survey article^32^. In the following, we list and analyze previous methods related to our proposed method.

Li et al.^33^ proposed first to train a model using clean data and then utilize the learned model to guide the learning process of a new model on the full dataset, which contains label noises. Tanaka et al.^34^ proposed to rectify labels while learning the DNN model parameters. An interchanged learning phase was designed where one training epoch asks the model to learn from the current labels, and the next training epoch uses the learned model to modify some of the labels the model cannot fit well. Zheng et al.^35^ trained a group of diverse deep neural networks using manually labeled (clean) labels, then they utilized the trained networks to generate pseudo-labels on unlabeled data. Manually labeled and network-labeled data is then combined to train a new model. In order to handle potential noisy labels in the network-generated labels, a random-fit and NN-fit algorithm was designed, which allows the under-trained network to choose which label to fit. Different from^34^, label distribution was better modeled by training a diverse set of neural networks, which helps avoid over-fitting. Han et al.^36^ generated prototype samples from the dataset and utilized the prototypes to correct some of the incorrect labels. Bai et al.^37^ compose a DNN model into multiple parts and control the early stopping timings for each part. This is based on the observation that earlier layers are often more robust than the later layers when learning from noisy labels. Early stopping helps prevent the network from over-fitting noises in the labels. Via such fine control of early stop w.r.t layers, this work achieves good performance in learning from noisy labels.

## Analyses on learning with noisy labels

To successfully utilize noisy labeled data for training a DL model, a crucial factor lies on whether any negative effects introduced by noisy labels can be minimized; meanwhile, the potential merits of using additional data (with noisy labels) can be maximized. As described above, many recent methods build on top of the original pseudo-label ideas, proposing new methods that aim to learn from noisy labels. Below, we give our analysis of the types of errors in the noisy labels, core mechanisms to handle these errors, and guidelines on how to design a DNN model with proper learning objectives and training procedures to achieve a successful learning procedure when dealing with noisy labels.

### Systematic and non-systematic errors

In this work, we utilize ants’ motion to generate pseudo-labels. This assumes any moving object in the videos is an ant object. But, occasionally, other small objects would move in the videos. The motion-based foreground detection algorithm (see “Detecting ants using motion-based foreground detection algorithms” section for details) cannot distinguish between ants and other insects. Thus it would treat all the moving objects as ants. These errors are systematic. On the other hand, winds occasionally cause objects to move in the scenes; in some frames, objects are moving, and in most cases, those objects are stable, and the labels would be assigned differently for the same objects. Thus, these errors are non-systematic.

More formally, we consider a group of *n* training samples $X=\{x_1,x_2, \dots , x_n\}$, $Y=\{y_1, y_2, \dots , y_n\}$, where $x \in {\mathbb {R}}^{w\times h \times c}$, and $y \in {\mathbb {R}}^{w'\times h' \times p \times q \times k}$. That is, an input image with width *w*, length *h*, and *c* number of channels, and its corresponding annotation is represented as a tensor with $w'$ width, $h'$ height, and *p* number of configurations of the bounding box’s width and *q* number of configurations of the bounding box’s height. *k* is the number of object classes in the detection problem. For semantic segmentation (a dense pixel classification problem), the above output is reduced to ${\mathbb {R}}^{w'\times h' \times k}$. To convince of the below analysis, we assume the problem is predicting whether the center pixel of a sample $x_i$ is a center of a target object. Hence the output $y_i$ is further reduced to ${\mathbb {R}}^{k}$. Suppose a subset of the samples are labeled incorrectly, and an indicator array $ind={b_1, b_2, \dots , b_n}$, where $b_i=1$ means the $y_i$ is correct, and $b_i=0$ means $y_i$ is incorrect. Note that in network training, the values for this array are unavailable. We consider an error in the label for sample *i* is a systematic error, if $b_i=0$, and for any sample $x_{i^*}$ that is visually similar to sample $x_i$ (according to a perceptual measure $\zeta$, that $\zeta (x_i)- \zeta (x^*) \le \epsilon$) and $y_{i^*}=y_i$. On the other hand, for those samples with $b_i=0$, if there exists another sample $x_i^*$ in the training set that is visually similar to $x_i$ (according to a perceptual measure $\zeta$, that $\zeta (x_i)- \zeta (x^*) \le \epsilon$), but $y_i^* \ne y_i$. Then we consider the errors of $y_i$ for a sample $x_i$ as a non-systematic error.

### Handling non-systematic errors

Shared masks/convolutions allow a convolution-based DL model to correct non-systematic errors. Suppose a group of convolution layers in a DL network works on a sample $x_A$, whose pseudo-annotations suggest an ant in this sample, $y^A = 1$. Suppose that there is another sample $x^B$ that is visually similar to the sample $x^A$ (that is $\zeta (x^A)-\zeta (x^B)\le \epsilon$, but pseudo-annotations suggest that no ant appears in the sample B ($y^B = 0$). Although these two samples are visually similar, their supervision signals provide significantly different suggestions. That is, the annotations of either sample A or sample B are less accurate or wrong. Since the parameters of the convolutions are shared across all the image areas (i.e., location independent) and the functions built by the convolutional layers are continuous, making the DL model fit both samples A’s label and sample B’s label challenging. Handling non-systematic errors is then transferred to the problem/task of preventing over-fitting. In “Detecting ants using motion-based foreground detection algorithms” section, we utilize motion-based foreground detection algorithms to generate pseudo-labels for ants detection task. In “Learning from algorithm-generated annotations” section, we give details of the training pipeline for learning from the algorithm-generated annotations.

### Handling systematic errors

Systematic errors are consistent for samples with similar appearance. Namely, a cluster of samples given the same wrong label from a labeling process. If no additional information (supervision) is available, rectifying this type of error is challenging. A potential way to tackle this problem is to generate multiple versions of pseudo-labels and select the most plausible label for each sample to fit. Generating multiple versions of pseudo-labels aims to break the consistent labeling pattern from a single labeling resource. Unless errors are consistent across all the versions of the pseudo-labels, there is a chance to correct some of the errors via a label selection procedure. In “Learning from multiple labeling sources” section, we describe the procedure of learning from multiple pseudo-annotations. In the case where all the labels provide wrong suggestions for a certain type of object (samples), manual annotations would be required to rectify such errors. “Using both algorithm-generated annotations and human annotations for network training” section describes how we perform joint learning using the algorithm and human-labeled data.

## Method

### Detecting ants using motion-based foreground detection algorithms

Given the fact that the background in an ant surveillance video is often relatively stable through time, our foreground extraction algorithm aims to extract moving objects in the video. In many biological studies, the background is usually static, and the moving objects in videos are usually the objects of interest in the studies (e.g., ants). Thus, foreground extraction algorithms are well suited to our dynamic ant problem to produce some preliminary detection results for the subsequent DL network training.

Given a video of *N* image frames, $X=\{x_i, i=1, 2, \ldots , N\}$, a foreground extraction algorithm decomposes each frame $x_i$ into two maps, $x_i^{bg}$ and $x_i^{fg}$, where $x_i^{bg}$ is for objects that stay static throughout the image sequence (i.e., background), and $x_i^{fg}$ is for objects that move through time (i.e., foreground). There is a rich set of methods and literature for algorithmic foreground extraction (e.g.,^38, 39^). Here, we describe a straightforward but effective one, which we also use in our experiments (denoted as FE-1).

Assume that each frame $x_i$ in a video is of a fixed size. For every pixel location (*p*, *q*), across all the *N* frames in the video, we compute the mean pixel value for this location ${\overline{x}}_{p,q}=\frac{1}{N}\sum _{i=1}^{N}(x_{i,p,q})$ and its standard derivation ${\sigma }_{p,q}=\sqrt{\frac{1}{N-1}\sum _{i=1}^{N}(x_{i,p,q}-{\overline{x}}_{p,q})^2}$. We then determine whether a pixel $x_{i,p,q}$ of $x_i$ belongs to the foreground or background by a straightforward criterion: If $|{x_{i,p,q}-{\overline{x}}_{p,q}}|>T \times \sigma _{p,q}$, then ${x_{i,p,q}}$ belongs to the foreground (${y_{i,p,q}}=1$); otherwise, ${x_{i,p,q}}$ belongs to the background (${y_{i,p,q}}=0$). Based on the empirical rule^40^, *T* can be chosen as 2, 2.5, or 3. In our experiments, we set *T* as 2.5.

The above procedure runs in linear time in the number of pixels in the image sequence, and can capture the essential structures of the moving objects in the sequence. To highlight the instance-level information for the ant detection problem, we define a new class called “ant boundary” using a dilation operation based on the foreground detection results of the algorithm. This allows us to directly enforce separation among nearby or touching ants during network training. The boundary class is commonly applied to image detection and segmentation problems. Our detection problem is now associated with three classes: ant body, ant boundary, and background. Besides the algorithm illustrated above, one can apply other algorithms (e.g.,^38^) for generating additional annotations for every image sample. Each version of the annotations could be used to train a detection network (“Learning from algorithm-generated annotations” section), and all the trained networks could be further used for the deep ensemble learning step (“Learning from multiple labeling sources” section).

### Learning from algorithm-generated annotations

As discussed above, a convolution-based DL model with shared convolutions/masks is one of the keys to the success of our DL network training. To show this, we examine two popular DL networks, U-Net^22^ and DCN^23^, for detecting ants in videos trained by noisy algorithm-generated annotations. Such a neural network mainly utilizes convolution operations (with batch normalization and ReLU), and its convolution kernels are shared across all image areas and are location insensitive.

Given image frames $x_i$, $i=1,2,\ldots , N$, and their corresponding algorithm-generated annotations $y_i$, $i =1, 2, \ldots , N$. We create a deep neural network $\tau$ with randomly initialized parameters $\theta$. The overall training objective function is $\frac{1}{N}\sum _{i=1}^{N}{{\mathscr {L}}}(\tau _{\theta }(x_i),y_i)$, where ${\mathscr {L}}$ is the spatial cross-entropy loss. We aim to minimize the objective with respect to the parameters $\theta$ in the network $\tau$. Following standard practice, we use the Adam^41^ optimizer to train the model with a batch size set as 8. The learning rate is set as 0.0005 for the first 30000 iterations and 0.00005 after 30000 iterations.

Suppose there are *K* algorithms to generate annotations. We repeat the above procedure *K* times to train *K* number of deep neural networks, each is supervised by annotations generated by one of the algorithms. Then we precede the final stage of our framework, that is, learning a single ensemble model from the *K* algorithm-trained neural networks.

### Learning from multiple labeling sources

Ensemble learning has been well studied before the deep learning era. It is commonly known that combining a group of diversely trained weak classifiers can often improve the prediction performance. Diverse base learners are essential for the success of ensemble learning. In this section, we aim to learn a new deep neural network from base neural networks that are trained by different algorithm-generated annotations. One can consider this as an ensemble learning problem, but the difference is that our learned model is a complex deep new network instead of a simple aggregator (e.g., majority voting or averaging). One can also view this task as a knowledge distillation problem that distills knowledge from multiple network resources. In either language, the task can be formally defined as follows. Given images $x_i$, $i=1,2,\ldots , N$; a group of diversely trained deep neural networks $\tau _1, \tau _2, \ldots , \tau _K$, train a new network $\tau _{final}$.

We propose a probabilistic version of the Random-NN-Fit (random-fit and nearest-neighbor-fit) algorithm^35^ for this deep ensemble learning task. We name the new algorithm as “**Probabilistic NN-Fit**”. Its goal consists of training a neural network while selecting which annotations to fit during every training iteration. The original Random-NN-Fit algorithm creates a two-stage training pipeline, where the first stage trains the network with uniformly randomly selected annotations, and the second stage trains the network by picking the annotations in favor of the under-trained network. Instead of exactly choosing the nearest neighbor to fit, in one iteration and for one image sample, our Probabilistic NN-Fit algorithm assigns each of its annotation versions a probability score that describes the chance that version would be selected for training. These scores are inversely related to the differences between the predictions of the current network and the annotations. A random sampling process is then applied to select an annotation version for each image sample for network training. Pseudo-codes of the Probabilistic NN-Fit is detailed in Algorithm 1.
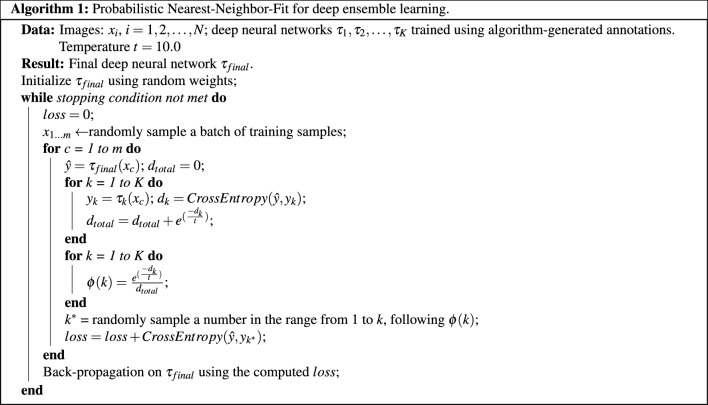


### Using both algorithm-generated annotations and human annotations for network training

When human-annotated images are available as training samples, it is reasonable to make use of both human-annotated (HA) and algorithm-annotated (AA) images for the DL network training. It should be noted that algorithm annotations and human annotations can be of different styles and nature, and thus directly mixing human annotations and algorithm annotations when training a DL model could cause confusion, especially for the decoding part of the model. We show here that our method is capable of utilizing these two types of annotations with only some minor twist of the DL model architecture.

**Figure 3:**
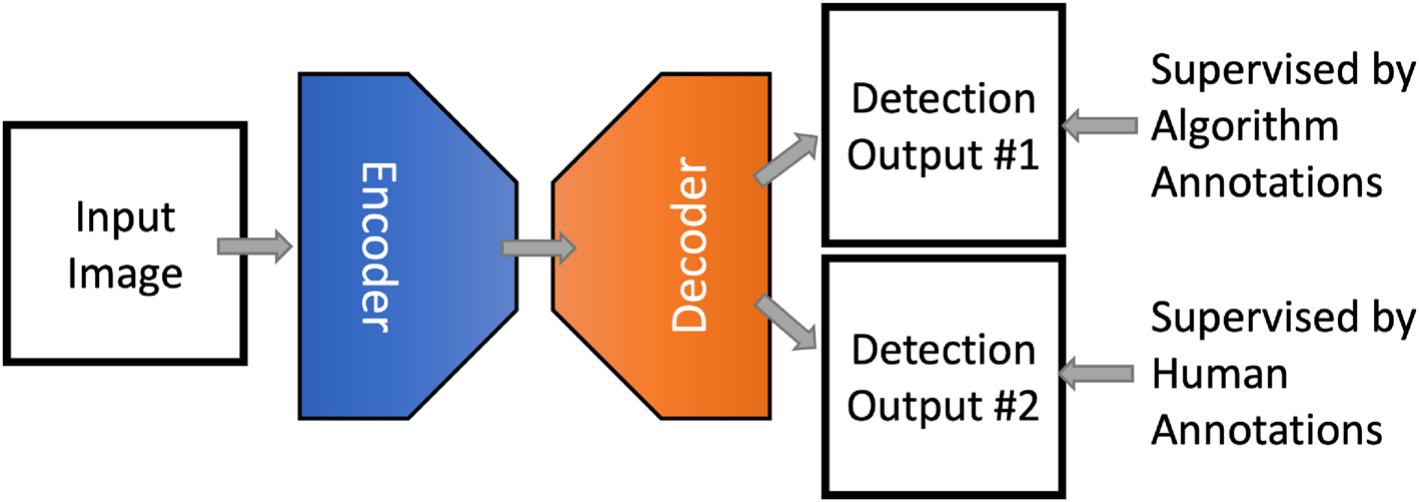
Training an encoder-decoder based deep neural network using images with algorithm-generated annotations and images with human annotations (when available).

We use a common U-Net model for illustration here. Different from those models that have only one output, we modify the deep neural network to give two outputs: output #1 is trained using images with algorithm-generated annotations and output #2 is trained using images with human annotations (see Fig. 3). We choose to have the output split near the end of the model for the following reasons: (1) this allows the AA branch to share the entire encoding backbone with the HA branch; (2) splitting near the end of the model output causes only a minimal increase in the model size, introducing only a few more convolution layers. Details are presented in Algorithm 2 and Fig. 3.
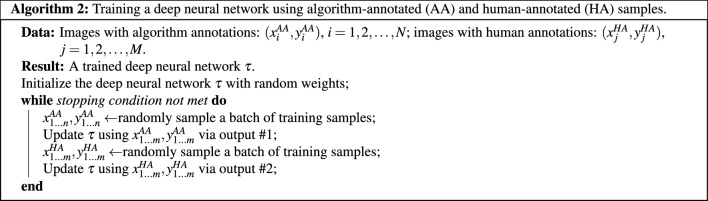

For the case in which both algorithm annotations and human annotations are used for training, there are two options for using the two output branches of the model during test: (1) using only the branch (output #2) that is directly trained with human annotations; (2) mixing the two outputs to generate the final detection results. We choose and recommend the second option in practice. Specifically, we add the two output tensors to obtain the probability map and then apply a normal *argmax* operation on top of it. As for the case in which only algorithm-annotated images are available, only output #1 is used for generating results in both training and test.

## Experiments

### Dataset

82 video clips (1080p) of ant motion were captured under natural conditions in a tropical rain-forest at night. The training set contains 65 videos (18,160 images) and the test set contains 17 videos (2188 images). We manually annotate all images, making the center point of every ant. Based on different experimental settings, part/all/none of the human annotations in the training set are made available to the DL network training.

### Evaluation metric

Since the human annotations that we use for the experiments mark the center point of each ant, the region-overlap type criterion is not suitable when computing matching between ground truth and the results of the DL model. We use a distance based criterion when computing matching between ground truth and the model prediction results. If an ant’s center point of the ground truth and a predicted ant center point are spatially close enough (e.g., $\le$ 5 pixels), we treat them as a potential matched pair. The overall matched pairs between ants in a ground truth image and detected ants by the model are computed using a maximum bipartite matching algorithm (also widely used to handle “points” for evaluation in contour detection^42^). The maximum bipartite matching is similar to a nearest neighbor based matching heuristic, but it is more systematic and accurate since it solves the matching problem with global optimality. After computing the matching between ground truth and the model predictions, we calculate the precision, recall, and $F_1$ score for the ant detection as in common practice.

### Scenario-1: using no human annotations

**Figure 4:**
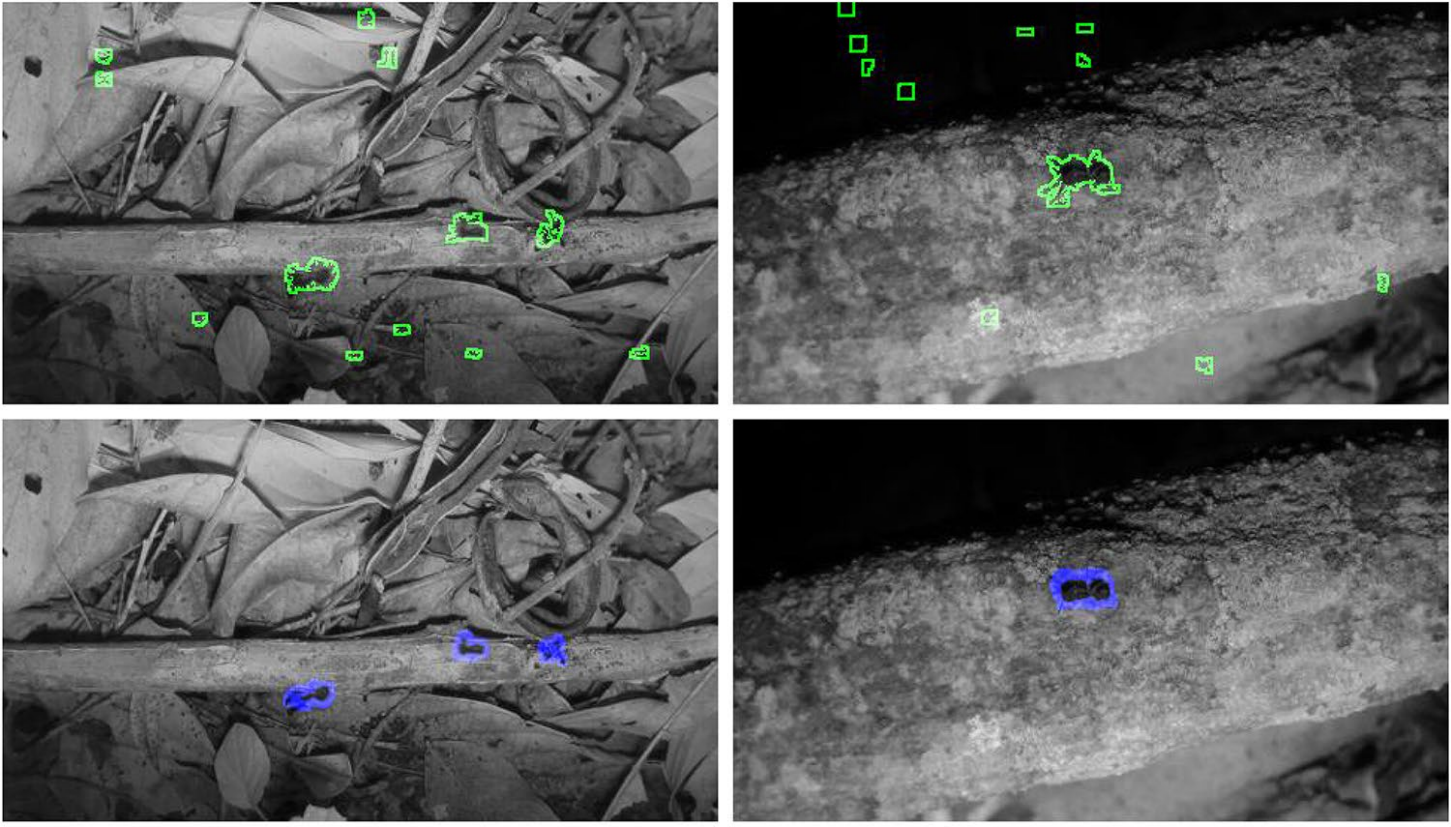
Top row: visualization of ant detection results from the FE-1 (foreground extraction) . Bottom row: visualization of ant detection results obtained from the U-Net trained using the FE-1’s annotations.

In this scenario, we train a detection network using only algorithm annotations. FE-1 is a simple algorithm based on basic pixel statistics (presented in “Detecting ants using motion-based foreground detection algorithms” section), and 3-term decomposition^38^ (dented as FE-2) is an algorithm based on low-rank matrix decomposition for foreground detection in videos. We believe that these are two representative foreground extraction algorithms for our ant detection problem. We use a foreground extraction algorithm (e.g., FE-1 or FE-2) to generate pseudo-annotations for all the images in the training set. The raw images and generated pseudo-annotations are then used to train a DL based detection model (e.g., U-Net^22^ or DCN^23^). Table 1 shows that our method can significantly improve the results from the initial results provided by the foreground extraction algorithm. Both FE-1 and FE-2^38^ have a relatively high recall score. This is because ants have some movement most of the time in the videos and foreground extraction algorithms are designed to be sensitive to moving objects. On the other hand, camera movement and other moving subjects (not ants) in the videos lead foreground extraction to produce false positives; thus, the precision is low for these algorithms. In Fig. 4, we give visual comparisons between results of a foreground extraction algorithm and a U-Net model (trained solely on the annotations of the same algorithm). We can see that the network training procedure successfully removes many false positives while keeping most of the true positives.

**Table 1:** Performance of ant detection on the test set.

Model	Precision	Recall	$F_1$ score
FE-1 (pixel statistics)	0.2836	0.7841	0.4165
FE-2 (3-term decomposition^38^)	0.1319	0.8282	0.2276
LFAGPA (Learn from FE-1):			
$\hbox{DCN}_{\mathrm{FE-1}}$	0.7292	0.7171	0.7231
$\hbox{U-Net}_{\mathrm{FE-1}}$	0.7192	0.7291	0.7241
LFAGPA (Learn from FE-2):			
$\hbox{DCN}_{\mathrm{FE-2}}$	0.6327	0.5671	0.5981
$\hbox{U-Net}_{\mathrm{FE-2}}$	0.5933	0.5945	0.5938

All the network training supervision signals are generated by algorithms. No human annotations are used in training the DCN and U-Net.

In Table 2, we show the ensemble learning results for simple average ensemble, Random-NN-Fit^35^ and the proposed probabilistic NN-Fit. Clear improvement is achieved using probabilistic NN-Fit when combining networks in varied architectures and trained by different annotations.

**Table 2:** Learning from networks with different architectures and trained with different annotations.

	$\tau _{base}:\hbox{U-Net}_{\mathrm{FE-1}}$			$\tau _{base}:\hbox{U-Net}_{\mathrm{FE-2}}$		
$F_1$ score	AE	NN-Fit^35^	DE (ours)	AE	NN-Fit^35^	DE (ours)
$\tau _{base}:\hbox{DCN}_{\mathrm{FE-1}}$	0.7453	0.7538	**0**.**7679**	0.7331	0.7412	**0**.**7562**
$\tau _{base}:\hbox{DCN}_{\mathrm{FE-2}}$	0.7280	0.7352	**0**.**7494**	0.6232	0.6410	**0**.**6605**

AE: Average Ensemble, DE: Deep Ensemble (Probabilistic NN-Fit). DCN was used for constructing the final model in NN-Fit and DE. Significant values are in bold.

### Scenario-2: using a small amount of human annotations

**Figure 5:**
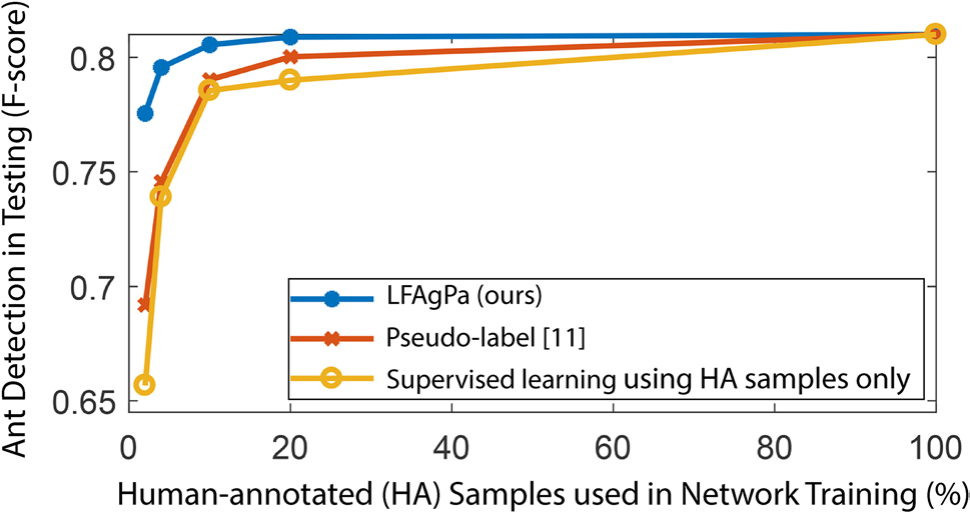
Ant detection performance for models trained using human-annotated and algorithm/DL-model annotated images. When using *k*% of human-annotated images, our method or the pseudo-label method^24^ utilizes the remaining $(100-k)$% images with automatically generated annotations for network training.

When human-annotated images are available for network training, we make use of them together with algorithm-annotated images for DL network training. For comparison, we consider the following training data proportion settings: 2% human annotation (HA) + 98% algorithm annotation (AA), 4% HA + 96% AA, 10% HA + 90% AA, 20% HA + 80% AA, etc. DCN is used for the experiments here. In Fig. 5, we show our method significantly improves the detection performance when utilizing AA images and performs considerably better than a widely used semi-supervised learning method^24^. Compared to the model trained using training images with $100\%$ HA, we achieve the same level of performance using only $10\%$ human-annotated training images. To support the analysis in “Using both algorithm-generated annotations and human annotations for network training” section, we further compare the model version illustrated in Fig. 3 with the model without the two-output design (i.e., single output). In Table 3, we demonstrate that the two-output design yields considerably better results than the simple single output design.

**Table 3:** Comparing the models with and without the output branching design.

Model	HA (%)	AA (%)	Precision	Recall	$F_1$ score
Using Full HA	100	0	0.8760	0.7520	0.8092
w/o output branching	2	98	0.8383	0.7006	0.7633
With output branching	2	98	**0**.**8482**	**0**.**7142**	**0**.**7754**
w/o output branching	10	90	0.8321	0.7493	0.7885
With output branching	10	90	**0**.**8631**	**0**.**7621**	**0**.**8090**

FE-1 generates AA. Significant values are in bold.

## Conclusion

Automatic tracking of moving objects in biological videos provides many new opportunities to study the individual and group behaviors of animals. Deep learning based detectors are very powerful, but they often require a large amount of manually annotated images in training. This paper demonstrated the feasibility of using algorithms automatically-generated annotations for training deep neural networks. More particularly, our proposed LFAGPA method can effectively train deep neural networks without using any human annotations, and by utilizing a small amount of human-annotated samples, LFAGPA can train a deep neural network to achieve the same detection performance as the model trained using full human annotations. LFAGPA provides a new practical way to tackle video detection problems in large-scale biological studies.

## Supplementary Information

Supplementary Information 1.

Supplementary Information 2.

## Data Availability

The dataset used in this study can be accessed by visiting this link https://drive.google.com/drive/folders/1WM_m-PPUcJoVj2gzZni99OkaJG1W1q9_?usp=sharing.
