# Supplementary material for: Learning from algorithm-generated pseudo-annotations for detecting ants in videos
Source: Sci Rep. 2023 Jul 18;13:11566. doi: 10.1038/s41598-023-28734-6 (PMC10354180; doi:10.1038/s41598-023-28734-6)
Supplement: Supplementary file 2 — Supplementary Information 2. [file 41598_2023_28734_MOESM2_ESM.docx]

**Supplementary Materials for “Learning from Algorithm-Generated Pseudo-Annotations for Detecting Ants in Videos”**

1. Visualization of Ants Detection Results (double click to view the video):


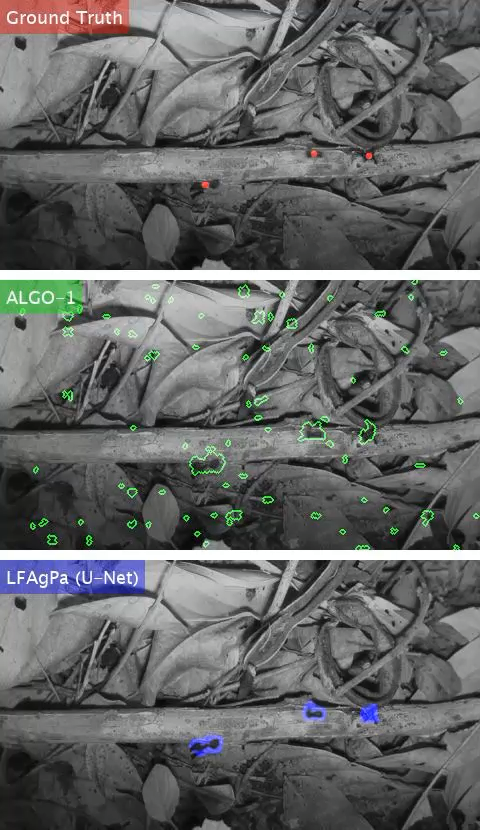
­­

Ground truth detections (visualized in red dots).

The detection results from a foreground extraction algorithm (visualized in green contours).

The detection results from a deep neural network (U-Net) trained using the results from the foreground extraction algorithm (visualized in blue contours).
